# Modularity analysis based on predicted protein-protein interactions provides new insights into pathogenicity and cellular process of *Escherichia coli *O157:H7

**DOI:** 10.1186/1742-4682-8-47

**Published:** 2011-12-22

**Authors:** Xia Wang, Junjie Yue, Xianwen Ren, Yuelan Wang, Mingfeng Tan, Beiping Li, Long Liang

**Affiliations:** 1 State Key Laboratory of Pathogen and Biosecurity, Beijing Institute of Biotechnology, Beijing 100071, China

## Abstract

**Background:**

With the development of experimental techniques and bioinformatics, the quantity of data available from protein-protein interactions (PPIs) is increasing exponentially. Functional modules can be identified from protein interaction networks. It follows that the investigation of functional modules will generate a better understanding of cellular organization, processes, and functions. However, experimental PPI data are still limited, and no modularity analysis of PPIs in pathogens has been published to date.

**Results:**

In this study, we predict and analyze the functional modules of *E. coli *O157:H7 systemically by integrating several bioinformatics methods. After evaluation, most of the predicted modules are found to be biologically significant and functionally homogeneous. Six pathogenicity-related modules were discovered and analyzed, including novel modules. These modules provided new information on the pathogenicity of O157:H7. The modularity of cellular function and cooperativity between modules are also discussed. Moreover, modularity analysis of O157:H7 can provide possible candidates for biological pathway extension and clues for discovering new pathways of cross-talk.

**Conclusions:**

This article provides the first modularity analysis of a pathogen and sheds new light on the study of pathogens and cellular processes. Our study also provides a strategy for applying modularity analysis to any sequenced organism.

## Background

Most cellular processes are carried out by groups of physically interacting proteins. Protein-protein interactions (PPIs) are at the heart of biological activities. A complete and reliable interaction map representing the specific binary interactions within a cell would provide a significant platform for understanding many biochemically relevant processes.

Several high-throughput experimental methods - such as pull down [1], immunoprecipitation [2], two-hybrid system, protein chips [3,4] - have been developed to detect PPIs among all the proteins encoded by a genome. While the data from these experimental approaches have been useful to biologists, there are several shortcomings. In particular, the results from high-throughput interaction mappings have low accuracy, and even reliable techniques can generate many false positives when applied genome-wide. Estimated error rates of high-throughput interaction results range from 41 to 90% [5,6]. Detecting experimental interactions is also labor-intensive and costly, in part because the number of possible PPIs is very large.

Computational methods provide a complementary approach to detecting PPIs and extending protein interactomes. A variety of computational methods have been applied to observe or predict the PPI networks in biological systems. These methods enable one to discover novel putative interactions and often provide information for designing new experiments for specific protein sets. The computational approaches to in-silico prediction can be classified into several general categories: genomic scale approaches [7], sequence-based approaches [8,9], structure-based approaches [10,11], protein domain-based approaches [12], learning-based approaches [13], and network-topology-based approaches [14]. Among these, certain domain-based prediction methods have shown great sensitivity and specificity to experimental PPIs, including MLE (Maximum Likelihood Estimation) [15] and MSSC (Maximum Specificity Set Cover) [16]. These approaches infer potential DDIs by relying on a training set of PPIs, and then use these potential DDIs to predict PPIs in testing sets.

However, obtaining networks of PPIs is not the final target. A major challenge is how to manage and analyze the huge number of data on PPIs. It has been reported that the metabolic networks of 43 distinct organisms are organized into many small, highly connected topological modules that combine in a hierarchical manner into larger, less cohesive units [17]. A module of a PPI network may represent a protein complex, or a group of proteins participating in the same cellular process. The prediction and analysis of PPI modules will aid us in elucidating the basic mechanisms of biological activities, while modularity analysis of the PPIs of pathogens could give us a better understanding of their pathogenicity.

Cluster analysis is an obvious choice of method for extracting functional modules from networks of PPIs. Clustering can be defined as the grouping of objects based on their shared discrete, measurable properties. A variety of clustering algorithms have been developed and successfully used in diverse fields. Recently, a systematic quantitative evaluation of the four most important clustering algorithms has been presented by Brohee and Van Helden [18]. The four algorithms were RNSC, MCODE, SPC, and Markov Cluster algorithm (MCL). Their results showed that the MCL algorithm was both remarkably robust to graph alterations and superior in the extraction of complexes from interaction networks.

The bacterium *Escherichia coli *O157:H7, which causes diarrhea and hemolytic uremic syndrome (HUS), is a worldwide threat to public health and has been implicated in many outbreaks of hemorrhagic colitis. The death rate for infected populations is between 5 and 10 percent. Currently, there is no effective method for curing or preventing infection. In 2001, the U.S.A. and Japan published the genome sequences of the EHEC 0157:H7 EDL933 and Sakai strains, respectively, which made genome-scale research on O157:H7 possible [19,20].

Several papers concerning modularity analysis of *E. coli *have been published in recent years. Von Mering et al. integrated techniques of conserved gene neighborhood, gene fusion events, and common phylogenic distribution to find functional modules of *E. coli *K12. By comparing to known metabolic pathways, they discussed pathway extension and functional links among pathways [21]. Gerdes et al. gave a system level analysis of essential genes in *E. coli *MG1655 based on experimental results, and discussed these essential genes in topological modules [22]. Li et al. developed a four-step approach for genome-wide discovery of parallel modules from protein functional linkages. This approach recovers known parallel complexes and pathways and discovers new ones [23]. To date, however, there are no published papers referring to modularity analysis of pathogens, whose PPIs are under-studied.

In this paper, the EHEC O157:H7 Sakai strain is selected for further research. Our aim is to analyze the modularity of the pathogen *E. coli *O157:H7 PPI network, without any known experimental PPI data. We also want to see what can be interpreted about pathogenicity and cellular processes by the modularity analysis. First, a domain-based method was used to predict the PPIs of O157:H7. Then we used the Markov Cluster algorithm (MCL) and separated 172 modules out of the predicted O157 PPIs. After evaluation, we found that most of these modules were functionally homogeneous and biologically significant. One hundred and twenty-one modules were considered highly reliable and may provide directions for experimental research. Six pathogenicity-related modules were analyzed, some of which are new and deserve further experimental validation. After investigation of the relationships among modules, the modularity of cellular function and cooperative effects are discussed. In view of these modules, our analysis can provide a better understanding of cell function. Moreover, the predicted modules can provide possible candidates for biological pathway extension and clues for discovering new modes of cross-talk between pathways. Overall, these results provide the first modularity analysis of a pathogen and shed new light on the study of pathogenicity and cellular processes.

## Results

### Prediction of *E. coli *O157:H7 PPIs

**Figure 1:**
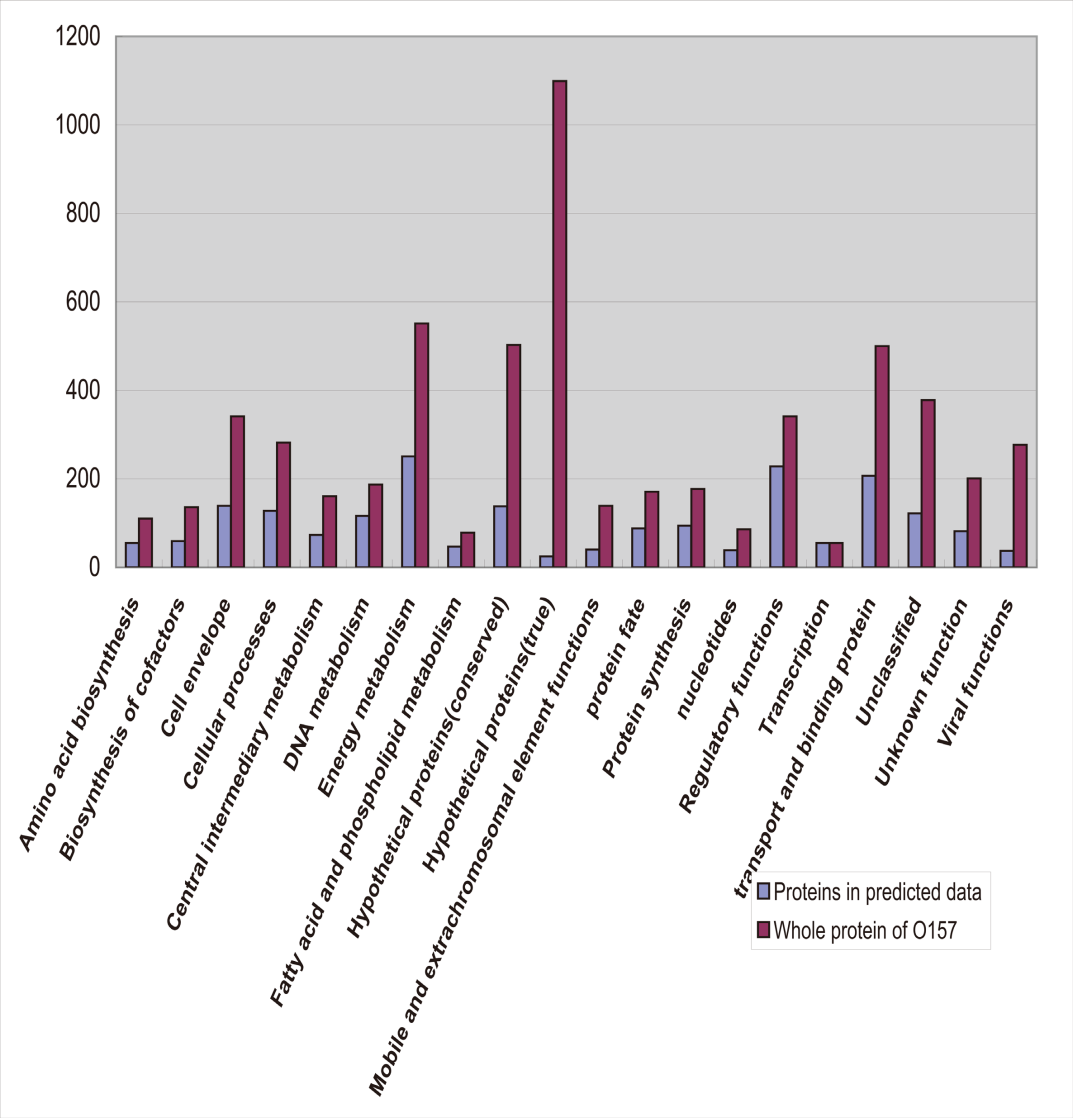
**Functional category comparison**. Y-axis represents the number of proteins. X-axis represents functional categories. Blue bars represent proteins in predicted data and red bars represent whole proteins from O157. We can see the greatest difference between the two is in the hypothetical proteins (true) category.

A domain interaction matrix is built using the MLE-MSSC method, based on 3722 credible protein interactions downloaded from the DIP database [24], which are validated by two or more experimental methods. All 5341 proteins of the *E. coli *O157:H7 Sakai strain are then scanned using the InterProScan program [25] to obtain the domains of these proteins. Among the O157 proteins, 2118 (39.4%) have domains that are present in the domain interaction matrix. In other words, 39.4% of the O157:H7 proteins can be used to predict PPIs. After computation, 24,995 PPIs involving 1701 proteins were predicted. Two post-processing steps were applied to eliminate directionally repeated interactions and self-interactions. The final dataset (additional file 1) contained 12,130 PPIs involving 1652 proteins, which are shown in Table 1 and in Additional file 2, Figure S1. We then used tools from the TIGR website to categorize the proteins predicted to have PPIs into 20 functional groups (Figure 1). From the figure, we can see that the two most different categories lie in "Hypothetical proteins (true)" and "Hypothetical protein (conserved)". So although about 60% of the proteins cannot be used to predict PPIs, a significant number of these proteins belong to hypothetical proteins, which have no clear functions and may not even be expressed. This could reduce the false negative effect caused by our PPI prediction method.

**Table 1:** Predicted data of O157 protein interactions

	Proteins predicted	Total protein of O157	percentage	Interactions	Average PPIs per protein
**Raw data**	1701	5341	31.8%	24995	14.7
**Processed data**	1652	5341	30.9%	12130	7.3

### Evaluation of predicted PPIs

The reliability of our predicted PPIs is the basis of this research, as the module prediction and further analysis are all founded on these data. Although MLE and MSSC were proved in their original papers to show good sensitivity and specificity, we still adopted several other methods (below) to validate the reliability of prediction.

### Comparison of predicted PPIs with STRING database

Currently, there are too few experimental PPIs from *E. coli *O157 to evaluate our predictions. So we chose the STRING database, which collects experimental and predicted PPIs. The PPIs in the STRING database include direct and indirect (functional) associations, which are derived from four sources: genomic context, high-throughput experiments, co-expression, and previous knowledge.

We downloaded the PPI data from the STRING database and 548,828 PPIs of EHEC O157:H7 Sakai strain were selected, relating to 5201 proteins. After comparing our predicted PPIs with the STRING datasets, we found that 2478 (20.4%) of them overlapped. PPIs are dynamic, so the results of different assays or even the same assays at different times or under different conditions can vary. The overlap percentage here is not far from the ~25% overlap of the high-throughput experimental yeast interactome by Gavin [26] and Krogan [27] respectively. So we can conclude that our predicted PPIs overlapped well with the existing data in the STRING database.

### Topological analysis of predicted PPIs network

To evaluate the predicted PPIs, we also analyzed the topological structure of the predicted PPI network to see if it had the same characteristics as the PPI networks generated by experiments.

Yook et al. compared four available databases that represent the protein interaction network of *S. cerevisiae *and found that the yeast protein interaction network in each database shows scale-free topology and hierarchical modularity [28]. Li et al. analyzed three of the largest protein interaction networks of *S. cerevisiae, C. elegans *and *D. melanogaster *and also confirm the scale-free, small-world property [29].

**Figure 2:**
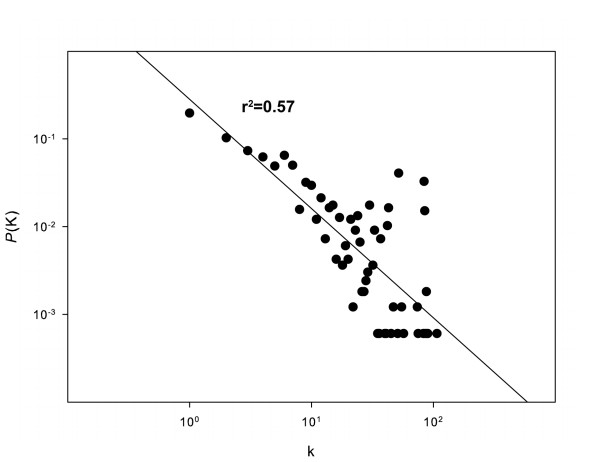
**Degree distribution of predicted PPI network**. Note that the degree distributions follow the power law, indicating it is a scale-free network. The degree exponent value is obtained from fitting to the function *P*(k) ~ k^-γ ^and is listed in table 2.

To investigate whether our predicted PPI network has a scale-free topology, the degree distribution was calculated. Degree k is the elementary character of a node, representing the number of other nodes linked to it. The degree distribution *P*(k) represents the probability that a node has k links. If the degree distribution follows the power law distribution, *P*(k) ~ k^-γ ^, then it means the network is scale-free [30]. The degree distribution of our predicted PPI network is shown in Figure 2. It follows the power law with a degree exponent of 1.7, indicating that the PPIs can be defined by a scale-free network. By randomizing the domain-domain interaction matrix, we repeated the prediction procedure and generated a random PPI dataset. Instead of being scale-free, the random PPIs dataset follows the Poisson distribution (additional file 3, Figure S1).

Clustering coefficients can reflect the network's modularity property. It is defined as C_i _= 2n_i_/k_i_(k_i_-1), where n_i _represents the number of direct links between the k_i _neighbors of node i. The clustering coefficient of a network is defined as the average value of all individual nodes. For the random network model, all the nodes share the same clustering coefficient: ${\text{C}}_{\text{rand}}=\text{p}=\frac{<k>}{N}$, where < k > is the average degree of the network and N is the total number of nodes. The relative clustering coefficient of the predicted PPI network is calculated and normalized by a random network of similar size [28]. The result is 54.3, which indicates that the nature of the clustering is far from random.

From topological structural analysis, we can see that our predicted PPI network has the same characteristics as those networks obtained by experiment (Table 2).

**Table 2:** Topological characteristics of experimental PPI networks [17] and predicted dataset of O157

Organism	No. of proteins	No. of interactions	Diameter of network	Relative clustering coefficient	Degree exponent
S. cerevisiae	4773	15461	4.2	50	1.8
C. elegans	4030	2638	4.8	35	1.6
D. melanogaster	20988	7068	4.5	24	2
Predicted dataset of O157	1652	12130	5.5	54	1.7

### GO distance analysis of predicted PPIs

The GO annotations [31] of *E. coli *O157:H7 Sakai strain are included in the Uniprot GOA annotation and thus are downloaded from the GO database. For each pair of proteins in our predicted dataset and random dataset, we computed the GO distance. The smaller the value, the more specific a category the two proteins belong to, so they are more likely to interact. The average GO distance of the predicted PPI dataset is 0.50, while that of the random dataset is 0.92. Student's t test shows that the predicted PPI dataset has a significantly low GO distance towards the random dataset (t = 82.5, P < < 10^-15^).

### Semantic similarity analysis of predicted PPIs

Besides the GO distance, the semantic similarity of GO terms is calculated to evaluate the predicted PPI dataset. For pairs of interacting proteins, GO terms in the "Biological Process" category are used to compute the semantic similarity score. The biological process aspect can be related to protein interactions, both physical and indirect (involved in same process). Of the 1651 proteins in the predicted PPI dataset, 1356 (82.1%) had the GO Biological Process annotation. The average semantic similarity of our predicted PPI dataset is 3.49, while the score of random set is 1.66. Student's t test shows that the predicted PPI dataset has a significantly high semantic similarity score (t = 52.6, P < < 10^-15^). The average scores of both datasets may be somehow higher, as we ignored interacting pairs lacking GO annotations in the "Biological Process" category.

Combining the comparison of STRING database, topological analysis, GO distance and semantic similarity analysis, we can tell that the predicted dataset is reliable enough for further analysis.

### Prediction of modules from PPI network

The Markov cluster algorithm (MCL) [32] is used to predict modules from the PPIs obtained above, with an inflation coefficient of I = 1.8. However, the modules predicted by the MCL algorithm have no overlapping components, while in real organisms some proteins exist in multiple complexes or participate in several cellular processes at the same time. We therefore identified the proteins shared between modules by a post-processing step. The shared proteins are shown in Additional File 4 Table S1; 172 modules were separated and the size distribution of the final predicted modules dataset (Additional File 5) is displayed in Additional File 6, Figure S1.

### Assessing the quality of derived modules

In order to determine whether the predicted protein modules are biologically significant and deserving of further research, we assessed the derived modules in three ways, described below.

### GO annotation analysis of the modules

We used the GO Biological Process annotation to evaluate the functional coherence of the modules predicted above. As we mentioned earlier, 1356 (82.1%) of the 1651 proteins in predicted PPI dataset had GO Biological Process annotation. We used these proteins as the background set. For each module and each GO term, we computed the enrichment of the term in the module versus the background set using a hypergeometric test and derived a P-value for the module. These P-values were further FDR-corrected for multiple testing. For each module, we chose the term that yielded the highest level of significance. Finally, the results showed that 121 modules (70.3%) had an enriched GO annotation (P < 0.01), which means that our predicted modules have good functional coherence. Details of the enriched GO terms and P-values of the modules can be seen in Additional File 7, Table S1.

### Comparison with known conserved protein complexes

Although there is no known database of the protein complexes of the *E. coli *O157:H7 Sakai strain, those complexes related to survival or reproduction will probably be conserved in other related organisms. We can find these modules from our predicted data, and then compare them with conserved protein complexes validated experimentally in other bacteria to evaluate the reliability of our predicted modules. To achieve this, we searched our modules in BOND (Biomolecular Object Network Databank) and in published references in PubMed. The results show that 55 of our predicted modules (32.0%) have complexes conserved in other bacteria, details of which can be seen in Additional File 8, Table S1. Three examples are given below.

Module 62 consisted of eight proteins (Additional File 9, Figure S1A), seven of which are components of the F_1_F_0_-type ATP synthase. This enzyme is of crucial importance almost ubiquitously because ATP is the common "energy currency" of cells [33,34], so it is conserved among almost all organisms. Module 114 (Additional File 9, Figure S1B) consisted of three proteins, RecO, RecF, and RecR. The RecFOR complex is also conserved in other organisms; it modulates the assembly and disassembly of RecA filaments on DNA and is essential in DNA recombination, repair and replication [35,36]. Module 126 (Additional File 9, Figure S1C) consisted of HypC, HypD, and HybG, which are also conserved in other bacteria. The complex functions in the assembly of the active site of the [NiFe]-hydrogenase enzymes [37].

### Comparison with KEGG pathway database

The KEGG pathway database [38] collects pathway data from metabolic processes, genetic information processes, environmental information processes and cellular processes. As we mentioned earlier, a protein module may not only represent a protein complex, but could also refer to a group of proteins participating in the same biological pathway or cellular process. So comparing our predicted module with KEGG pathways is useful for the evaluation. We adopted the following method to achieve this goal. First, we used Uniprot GOA annotation to annotate the GO Term (Biological Process) for each KEGG pathway and an enriched GO Term was obtained for each KEGG pathway. If more than two proteins in our predicted modules belonged to KEGG pathways, we selected the most overlapping KEGG pathway for further comparison. If the enriched GO term of both the module and the most overlapping KEGG pathway were the same, we deemed that the module was reliable; otherwise, we decided that it was not. Eighty modules have at least two proteins that belong to 92 KEGG pathways. Among these, 51 (63.8%) modules have an enriched GO term identical with the KEGG pathway. Because of the incompleteness and bias of the KEGG pathway data, we can infer that other modules that have no overlap with a KEGG pathway may have the same rate of reliability.

Combining GO annotation, comparison with conserved protein complexes and KEGG pathways, we can conclude that the predicted modules have good functional homogeneity and biological significance, which gives us confidence for further discussion. In all of the 172 predicted modules, 121 functionally significantly enriched modules identified by GO annotation analysis were considered highly reliable, which can give clues for further research.

## Discussion

### Predicted modules provide new information on pathogenicity

Two main factors contribute to the pathogenicity of EHEC O157:H7. First, the strain's ability to adhere and colonize, involving invasion, proliferation and resistance to the host immune system, makes it an effective pathogen. Second, the strain produces toxins, including endotoxin and exotoxin [39]. Among our predicted modules, we found six functional modules that contain known virulence factors and may relate to *E. coli *O157's pathogenicity.

### Novel module in cell adhesion

**Figure 3:**
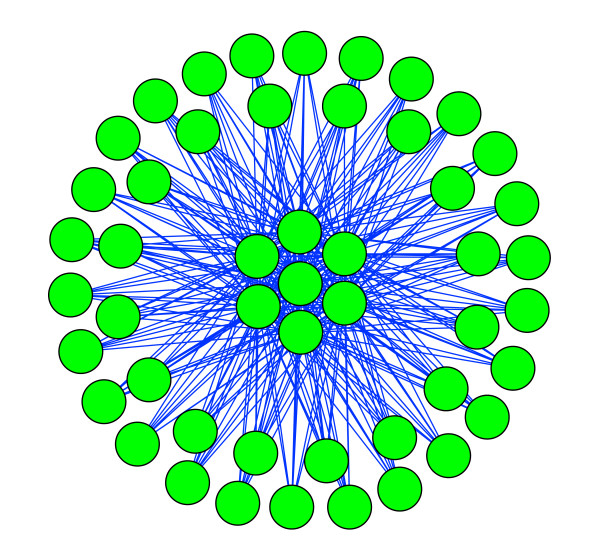
**Protein interaction map of module 6**.

Module 6 consists of 49 proteins (Figure 3), and from Figure 3 we can see that the topology of the protein interaction map from this module is very interesting. The 42 peripheral proteins only interact with the seven central proteins; neither peripheral proteins nor central proteins interact amongst themselves. The GO annotations of the 42 peripheral proteins are all "cell adhesion" (GO ID: 000715), while most of the proteins' annotations in NCBI are "putative fimbrial protein", including LPF (long polar fimbriae) [40] and Type-1 fimbriae [41,42], which were already experimentally validated as important virulence factors in adhesion processes of O157:H7. The NCBI annotations of the seven central proteins are listed in Table 3; all seven scanned by the InterProScan program contain a domain called "Penicillin-binding protein, transpeptidase fold" (IPR012338). We used TMpred to predict transmembrane regions of these proteins, and the results showed that six of the seven proteins have at least one membrane-spanning region, which suggests they are transmembrane proteins. We then predicted the signal peptides of the seven proteins by SignalP 3.0 [43], and the results revealed signal peptides in five of them, which means they are probably secreted proteins. These results provide the possibility that the central proteins have a cellular location allowing them to interact with fimbirae proteins, as fimbriae proteins are located in the outer membrane. This module has not yet been reported in any published reference. Further experimental validation and research is merited for this novel module.

**Table 3:** NCBI annotation, SignalP and TMpred prediction result of the seven central proteins

Protein ID	SignalP	TMpred	annotation
NP_312106.1	secreted	1	Hypothetical protein
NP_308697.1	secreted	2	D-alanyl-D-alanine carboxypeptidase fraction A
NP_310158.1	unsecreted	1	glutaminase
NP_312469.1	unsecreted	0	glycyl-tRNA synthetase beta subunit
NP_311137.1	secreted	1	hypothetical protein
NP_308453.1	secreted	2	hypothetical protein
NP_311048.1	secreted	1	penicillin-binding protein 7

### Landscape of O157:H7 iron acquisition system

**Figure 4:**
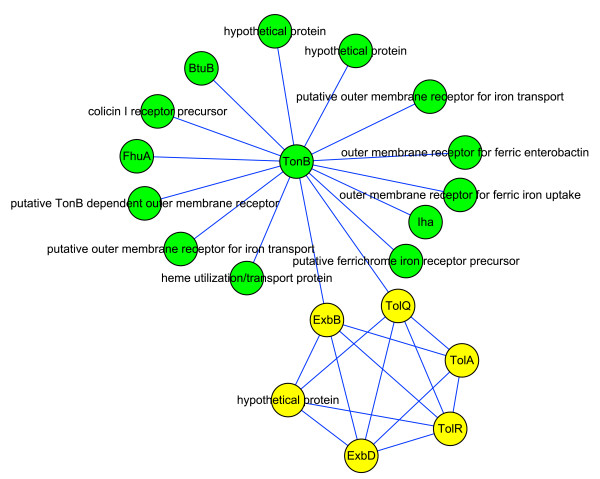
**Interaction map of modules associated with iron acquisition**. The green nodes belong to module 35, and the yellow nodes belong to module 75.

The protein interaction map in Figure 4 consists of two modules associated with iron acquisition. Iron is required by most living cells because of its diverse roles in numerous metabolic processes, including glycolysis, energy generation by electron transport, and DNA synthesis. Iron forms highly insoluble ferric hydroxide complexes, which severely limits its bioavailability for use by pathogens. However, invading pathogens must gain access to host iron for survival, so two systems have evolved to acquire it from the iron- or heme-chelating proteins of mammalian hosts. The first mechanism relies on siderophores, which are compounds of low molecular mass and enormous avidity for ferric iron [44]. The second method is the direct use of iron-containing complexes, especially heme proteins [45].

Importing both siderophores and heme proteins into pathogens requires outer membrane receptors and TonB-ExbB-ExbD import systems [46], which is the case in module 35 in Figure 4. Besides iron, the uptake of vitamin B12 also depends on the TonB system, and BtuB in Figure 4 is the outer membrane receptor for B12 [47]. The TolA, TolR, and TolQ elements in the yellow module share similarities with the TonB system and may contribute to the integrity of the cellular envelope [48].

Two proteins are annotated as "hypothetical protein" in the green module; the Blast result shows that they have high similarity to a TonB-dependent outer membrane receptor. We also used Signal P and Tmpred [49] to see whether they have signal peptides or transmembrane domains. Both of the proteins were predicted to have signal peptides, meaning that they are probably secreted proteins. One of them was predicted to have four possible transmembrane domains, while no transmembrane domains were predicted for the other. Further experiments are suggested to validate their roles as outer membrane receptors.

We can see from Figure 4 that there are multiple outer membrane receptors for iron, probably because iron is essential for O157; in this way the pathogen can increase its tolerance to mutations and ensure the continued import of iron. It has been reported that mutations in one of the outer membrane receptors do not affect iron acquisition, while a TonB mutant failed to use heme as an iron source or to utilize the siderophores, and showed reduced virulence [50,51]. Our predicted interaction map can explain this well, as TonB plays a hub-like role, such that mutation or deletion of it will destroy the network and thereby cause inefficient iron acquisition.

### Shiga toxin: the most important virulence factor of O157

**Figure 5:**
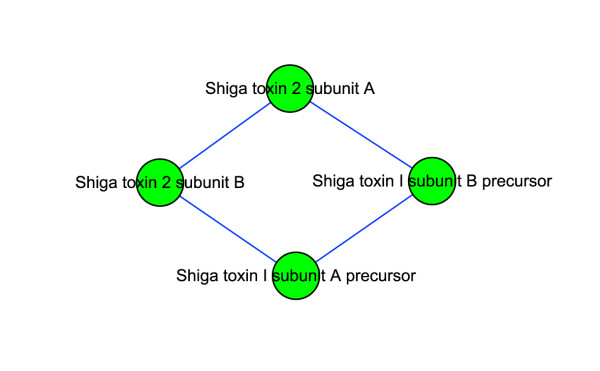
**Interaction map of module 101 related to Shiga toxin**.

Module 101 (Figure 5) contains four proteins. Stx (Shiga Toxin) is a key virulence factor of O157. It consists of two subunits, A and B, with the structure A_1_B_5_. *E. coli *O157 can produce Stx1 and Stx2. Stx1 exists internally, while Stx2 is secreted to the environment. Stx2 is about 1000 times more toxic to human renal microvascular endothelial cells than Stx1 [52]. Also, experimental support for the association between Stx2 and severe diseases was provided by Siegler et al., who compared the effects of Stx1 and Stx2 in a primate animal model of HUS [53]. The proteins in Figure 5 are subunits of Stx1 and Stx2. We can see that there are interactions between Stx1 and Stx2, perhaps because their subunits are similar (55% identity in amino acid sequence between the A subunits of Stx1 and Stx2), causing a false positive. It is also possible that in some periods they join to form a complex, and are split afterwards. If the latter hypothesis can be proven experimentally, then we can explain why organisms that produce both Stx1 and Stx2 are paradoxically less virulent than those that produce Stx2 but not Stx1 [54]. The interactions between Stx1 and Stx2 could prevent Stx2 from being secreted. Also, we might be able to design a drug to disable the splitting of Stx1 and Stx2, thereby effectively reducing the virulence of O157.

### Other pathogenicity-related modules

Module 88, displayed in Additional File 10, Figure S1A, contains Tir (Translocated Intimin Receptor), which is secreted by a type III secretion system (TTSS) and inserted into the host cell membrane as a receptor for intimin [55]. CesT is Tir's chaperone, and it directs Tir to TTSS ATPase EscN [56]. EscN functions in substrate recognition and in chaperone release from, and unfolding of, type III secreted proteins [57]. Module 115 has three proteins, shown in Additional File 10, Figure S1B: the α, β, and γ subunits of urease. The complex catalyzes the hydrolysis of urea into ammonia and carbon dioxide. Expression of urease could modify internal and/or surrounding anion concentrations, enabling O157 to survive in acidic conditions and perhaps contributing to its low infectious dose [58]. Module 130 consists of two periplasmic proteins, RseA and RseB, together with rpoE. RpoE is a sigma factor responsible for the transcription of genes for the cell envelope stress response. Under normal conditions, it is kept inactive by its interaction with the periplasmic proteins RseA and RseB. Under stress conditions, a protease in the periplasm cleaves the interaction between rpoE and the RseAB complex. Then rpoE is free to regulate the expression of genes associated with the stress response. Inactivation of rpoE diminishes bacterial survival and growth inside host macrophages, as it also regulates genes required for oxidative stress resistance [59].

From our study of the pathogenicity-related modules above, we can see that our modularity analysis of O157:H7 provided new information about the pathogen and clues for further experimental validation. This new information not only includes novel modules and speculations about the functions of new proteins, but also pathogenicity from the point of view of the module level. However, owing to false negatives in PPI prediction, other pathogenicity-related modules have not been shown here. With more complete and reliable experimental PPI data and more accurate in silico prediction methods in the future, the modularity analysis of pathogens will help us generate a better understanding of pathogenicity.

### Investigation of relationships among predicted modules

**Figure 6:**
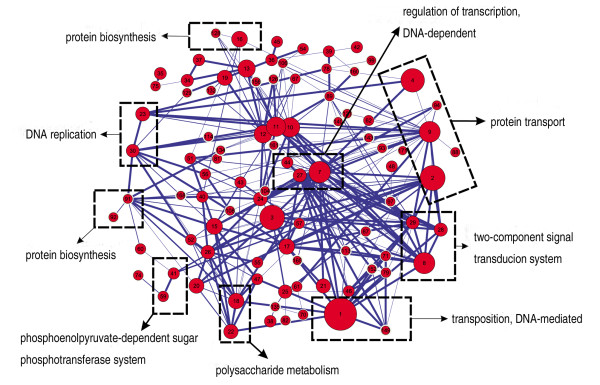
**Relationships among predicted modules**. Red node in the network represents a module. Node radius is proportional to the module's size. Node labels represent module number. Edges represent at least two PPIs among modules. Modules in dashed rectangle have the same GO Biological Process Term as each other.

In this study, 172 modules are divided by a mathematic algorithm from a complex PPI network shown in Additional File 2, Figure S1, while in the real organism they may have relationships, even with few or no interactions among them. It has been reported that complex cellular processes are modular and are accomplished by the concerted action of functional modules [60]. Therefore, to study cellular processes, it is of great importance to determine the relationships among the 172 predicted modules and their meaning. For this investigation, we considered three kinds of information: Modules were deemed related if (1) they have direct PPIs or overlapping components, (2) they have the same enriched GO term of Biological Process, since they might participate in the same cellular process, (3) they overlap with the same KEGG pathway and have the same enriched GO term as the KEGG pathway. These three kinds of information were then integrated and the results are listed in the supporting files. Figure 6 shows an example of integrating two of the kinds of information mentioned above. The relationships of the predicted modules provide a guideline for the discussion of cellular process below.

### Predicted modules demonstrate modularity of cell function

In the 21st century, research in cell biology is changing from molecular to modular, and from studying the function of only one gene or protein to investigating how a group of biological molecules functions as a module. The conception of modularity of cell function is that cell functions are carried out by different modules, which comprise many types of molecules. Through studying the relationships among modules, we found that our predicted modules demonstrate the modularity of cell function.

**Figure 7:**
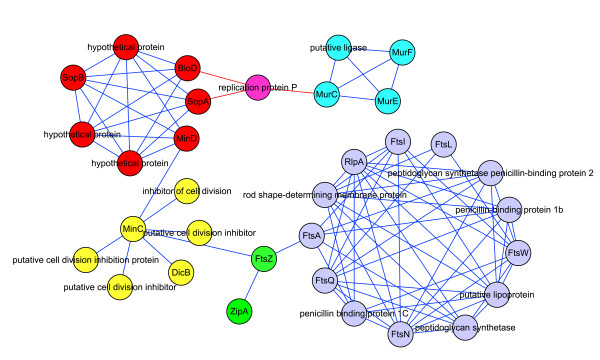
**Modules related to cell division**. Green nodes belong to predicted module 135, yellow nodes belong to module 70, red nodes belong to module 61, blue nodes belong to module 93, and purple nodes belong to module 38.

Figure 7 consists of five small modules related to cell division in O157. In the green module, FtsZ is a GTPase that forms a ring-like structure known as the Z-ring at the midcell boundary [61,62]. There are some similarities between FtsZ and tubulin, so it is not surprising that the Z-ring is a highly dynamic structure. ZipA is a stabilizing factor of the Z-ring [63], while proteins in the yellow module are destabilizing factors of the Z-ring [64]. SopA and SopB, in the red module, are related to the partitioning of the plasmid during cell division. After BLAST, we found that two of the three hypothetical proteins in the red module were highly similar to ParA and ParB, which are also related to partitioning of the plasmid [65]. MurF, MurC, and MurE, in the blue module, are associated with cell envelope biosynthesis [66]. The Fts proteins in the purple module assemble on the Z-ring in order, though their functions are still not clear. The other proteins in the purple module are related to cell wall biosynthesis [67].

Because of false negatives, there may be some cell division-related modules that have not been predicted, but our modularity analysis still gives a landscape that multiple modules of different functions coordinate to accomplish cell division. This coordination does not only refer to small modules interacting directly to form a large module. For example, in Figure 7, the interaction between modules may be because two modules share the same protein, though they may be separated by time or location. But when they finish their own functions, they still macroscopically coordinate to fulfill a more important cellular function. In our other predicted modules, we also found the phenomena of modularity of cell function, which is listed in Table 4.

**Table 4:** Numbers of modules related to other cellular functions in our predicted data

Cellular Functions	Number of related modules(module ID)
DNA repair	9 (66, 67, 81, 82, 86, 98, 108, 114, 119)
DNA binding	6 (1, 13, 25, 44, 56, 83)
Signal transducer activity	4 (8, 17, 28, 29)
Structural constituent of ribosome	7 (91, 92, 97, 122, 127, 128, 151)
Transporter activity	8 (2, 4, 14, 15, 75, 35, 94, 96)
Transcription factor (regulator)	7 (5, 7, 24, 27, 48, 74, 112)

There are still many problems to solve regarding the modularity of cell function, such as how other cell functions are modular, or what the relationship is among modules fulfilling the same cellular function or how they coordinate and assemble. Once these problems are solved, the era of synthetic biology will really arrive. We will be able to design and synthesize a functional module in the same way that we design complex electronic modules or chips for a computer. Moreover, we will be able to arrange different functional modules properly, so as to simulate the cellular environment or synthesize new life.

### Predicted modules show cooperative effects

Cooperativity among biological molecules, for example enzymes, is established beyond doubt. We investigate the relationships among our predicted modules and find that cooperativity also obtains at the module level. The effect of different modules with similar functions cooperating to fulfill a cellular process is described as a positive cooperative effect; if these modules perform opposite functions, then we call it a negative cooperative effect.

### Positive cooperative effect

**Figure 8:**
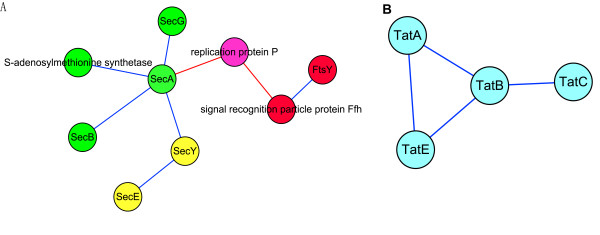
**Modules related to protein export**. A: Modules related to Sec translocation system. B: Modules related to the TAT system.

In Figure 8A, three predicted modules make up the Sec protein translocation system. This system is widespread in bacteria. The substrate proteins of the system have amino-terminal signal peptides, and they are transported in an unfolded state, which is largely driven by the energy released during ATP hydrolysis. SecYEG forms a complex and is likely to oligomerize to form a protein-conducting channel across the cytoplasmic membrane. SecA is an ATPase that provides energy for protein translocation [68]. The SecYEG-A complex constitutes a functional entity, and additional proteins are involved in protein translocation across the cytoplasmic membrane. SecB is a molecular chaperone, which binds to the mature portions of preproteins and facilitates their targeting to the translocation system via its affinity for SecA [69]. Ffh and FtsY, in the red module, are involved in another targeting route for precursor proteins that is mediated by the signal recognition particle [70]. Figure 8B shows predicted module 96, which corresponds to another protein translocation system: the twin-arginine translocation (TAT) system, which operates in thylakoid membranes and the plasma membranes of a wide variety of prokaryotes. In contrast to the Sec system, TAT has the unique ability to transport folded proteins through tightly sealed membranes, and it does not seem to require ATP hydrolysis at any stage of translocation [71].

We can see clearly that these four modules of two systems carry out similar functions and cooperate to fulfill the cell's protein export functions.

### Negative cooperative effect

In Figure 7, we have mentioned that the green module plays a role in forming a dynamic Z-ring structure at the midcell boundary and stabilizing this structure. Since the components in the yellow module play an opposing role, they are destabilizing factors of the Z-ring. These two modules together shift the equilibrium of FtsZ expression between an unassembled cytoplasmic pool and the assembled ring. The negative cooperative effect between these two modules ensures that the Z-ring only forms in the midcell instead of other places in the cell, so cell division is accomplished properly.

Of course, besides the cooperative effect, there might be other relationships among modules such as regulation, activation, deactivation and so on. But investigating these relationships requires other information and remains a great challenge.

### Predicted modules provide possible candidates for biological pathway extension

Biological pathways can be regarded as a collection of known relationships or reactions among biological objects (i.e., genes or gene products). However, knowledge about biological pathways is not complete, and known pathways are insufficient to cover all genes or gene products. In the case of humans, for example, about 3,000 genes are covered by the major biological pathway databases. The rest, and their relationships with other genes or gene products, remain to be explored [72].

In the previous assessment, we found modules that overlapped with the KEGG pathway and had the same enriched GO terms. In these modules, proteins not overlapping may be possible candidates for KEGG and may provide information for pathway extension. Two examples are given below.

**Figure 9:**
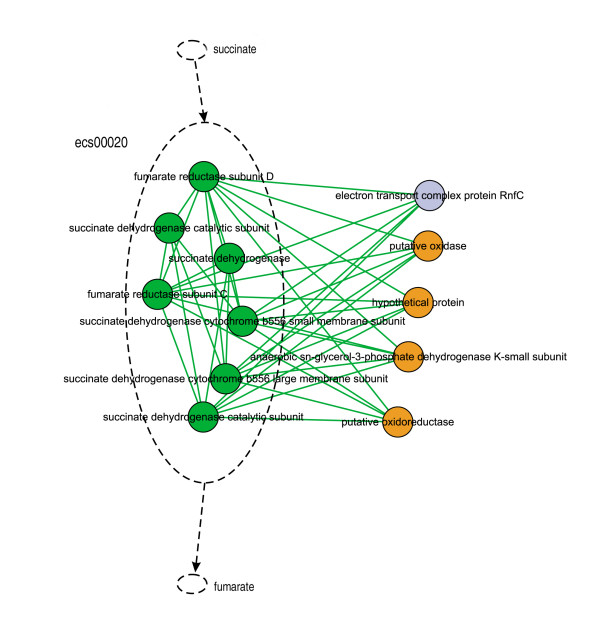
**Possible candidates for biological pathway extension**. Green cycles represent proteins overlapping with ecs00020; orange cycles represent candidate proteins that have no overlap but have the same GO term as the pathway.

There were 12 proteins in module 42 (Figure 9), seven (green nodes) of which had overlaps with KEGG pathway ecs00020: Citrate cycle (TCA cycle). In this pathway, these seven proteins form an enzyme (1.3.99.1) to catalyze the conversion of succinate to fumarate. Four (orange nodes) of the five remaining proteins had the same GO "Biology Process" term as these seven and the enriched GO term of the pathway. It is inferred that those four protein candidates might either be components of the enzyme or play roles in regulating its activity.

Module 4 consists of 53 proteins, 51 of which overlap with KEGG pathway ecs02010: ATP-binding cassette (ABC) transporters (Additional File 11, Figure S1). ABC transporters play an important role in bacteria, importing various nutrients required for survival in different niches and exporting substances toxic to the cell. Characteristically, ABC transporters have three components: a substrate-binding protein, a permease protein, and an ATP-binding protein. The NCBI annotations of the remaining two proteins in module 4 were "putative binding-protein dependent transport protein" and "putative transport system permease protein", respectively. This suggests that these two proteins are possible candidates for KEGG pathway extension and deserve further experimental validation.

From the two examples above, we can see that modularity analysis of O157:H7 has the capacity to provide possible candidates for, and facilitate research into, biological pathway extension.

### Predicted modules gave clues for discovering new cross-talk

Few pathways are isolated. Cross-talk among pathways links them into a complex network [73]. Such a network, with cross-talk linking distinct pathways, would confer on the cell a sophisticated ability to sense multiple environmental signals impinging upon it, thus providing a means of adapting or regulating its response to a particular range of effectors [74]. Major new challenges have arisen from attempts to identify cross-talk among pathways.

**Figure 10:**
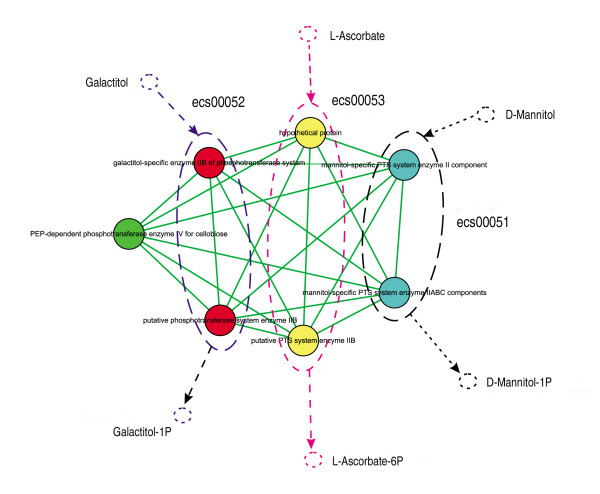
**Clues for discovering new cross-talk**. Blank dashes represent pathway ecs00051, while pink represents ecs00053 and purple represents ecs00052.

Module 65 (Figure 10) consists of seven proteins that all have phosphotransferase activity. From the figure, we can see that three pathways are presented in this module. They are ecs00051: "Fructose and mannose metabolism", ecs00052: "Galactose metabolism", and ecs00053: "Ascorbate and aldarate metabolism". Interactions among these pathways could be possible cross-talk. Some false-positives might exist, and experimental validation is needed for further identification of cross-talk. However, our modularity analysis has the ability to provide clues that may lead to discovering new cross-talk pathways.

### Concluding remarks

Modularity analysis of PPIs has become an important and challenging topic in life science. It will be instructive for scientists exploring the basic rules of cell processes or studying the mechanisms of pathogens. In this research, we integrated several bioinformatics methods for modularity analysis of *E. coli *O157:H7. We predicted PPIs inside the bacterium, then we derived predicted modules. Evaluation showed that these predicted modules were functional, enriched, and biologically reliable. One hundred and forty-four highly reliable modules were provided as directions for experimental research. Through our modularity analysis, pathogenicity-related modules can be found, which provide new information regarding the mechanisms of pathogenicity and enable us to study pathogens in a new light. Our predicted modules also suggest that some cellular functions are modular and give a more comprehensive understanding of cell functions. Cooperativity among modules was discussed. Moreover, our modularity analysis of O157:H7 provides possible candidates for the extension of biological pathways and clues for discovering new cross-talk between pathways. The method for modularity analysis provided in this study can be applied to other pathogens or any other organism of interest that has been sequenced.

The bottleneck of this method is its reliance on the prediction of PPIs. Because there are no available, experimentally validated data on PPIs for *E. coli *O157:H7, we adopted a domain-based bioinformatics method to predict such PPIs. Although this method has been successful and has some advantages compared to other predictive methods, we can see from the results that less than 1/3 of the proteins of O157 were predicted to have PPIs, which caused high false negative rates. False positives also exist in this incomplete set of PPIs. These problems limited later modularity analysis. We believe that with the increase of experimental data and the development of analytical methods, modularity analysis of all biological interactions will greatly facilitate research in life science and the development of synthetic biology.

## Materials and methods

### Datasets used

All datasets used in this study were downloaded in November 2007. DIP [24] provides 3722 creditable protein interactions, which are validated by two or more experimental methods. These 3722 PPIs were selected from 54,511 PPIs deposited in the DIP database referring to more than 200 organisms, including both non-pathogens and pathogens. Both direct (physical) and indirect (functional) associations are included among the 548,828 PPIs of *E. coli *O157:H7 used for overlap analysis and downloaded from the String database. The PPIs in String are derived from four sources: genomic context, high-throughput experiments, coexpression, and previous knowledge. Sequences for the proteins of *E. coli *O157:H7 are downloaded from the NCBI Refseq database in Fasta format. One hundred and twelve pathways of O157 are downloaded from the KEGG pathway database [38].

### Prediction of *E. coli *O157 PPIs

Maximum Likelihood Estimation (MLE) and Maximum Specificity Set Cover (MSSC) are both based on the Association Method (AM). These methods use currently available PPI data, and estimate the probabilities of domain-domain interaction observed in PPIs. Using the inferred domain-domain interaction, these methods can then predict previously unknown protein interactions. As MLE and MSSC modify AM in different and independent ways to improve accuracy, we combined these two methods to achieve a better result. In this work, the program InterProScan version 12.0 is adopted to scan domains of the creditable protein interactions from DIP. Then MLE and MSSC are used to build a domain interaction matrix for the prediction of PPIs. InterProScan was used again to scan domains of proteins of *E. coli *O157:H7 Sakai. The total matched pairs of the O157:H7 proteins were compared with the domain interaction matrix, and raw predicted PPI data were obtained. Two post-processing steps were applied to the raw PPI data. First, we eliminated directional repeats from the PPIs. Because the prediction program cannot predict weighted directional PPIs, directional PPIs are actually the same. Second, we eliminated self-interactions. The existence of self-interactions will generate single protein modules when the MCL algorithm is used for prediction. Although simple protein modules may represent homogeneous multimers, these would be difficult to analyze without additional information.

### Computation of GO distance

For any two interacting proteins, we calculate the Gene Ontology distance between them, taking into account all GO terms that are common to the pair and terms specific to each protein. Any two proteins can have several shared GO terms (common terms) and a variable number of terms specific for each other (specific terms). The GO distance between interacting proteins *i *and *j *is calculated using the Czekanowski-Dice formula [75]:

$${\text{D}}_{i,j}=\frac{|T_{GO}\left(i\right)\Delta T_{GO}\left(j\right)|}{|T_{GO}\left(i\right)\cup T_{GO}\left(j\right)|+|T_{GO}\left(i\right)\cap T_{GO}\left(j\right)|}$$

In this formula, *T_GO _*are the sets of the proteins' associated GO terms, while |*T_GO_*| stands for their number of elements and Δ is the symmetrical difference between the two sets. This distance formula emphasizes the importance of the shared GO terms by giving more weight to similarities than to differences. Therefore, for two genes that share no GO terms, the distance is 1, while for two proteins sharing exactly the same set of GO terms, the distance is 0. Based on this algorithm, some Perl programs were written to implement the calculation and analysis of GO distance in our study.

### Calculation of GO semantic similarity

We used an R based package, csbl.go http://csbi.ltdk.helsinki.fi/csbl.go[76], to calculate GO semantic similarity. The package can compute similarities for arbitrary numbers of genes and supports custom GO annotation tables. For pairs of interacting proteins, the semantic similarity of GO terms in the "Biological process" taxonomy is calculated on the basis of the Resnik measure.

### Prediction of PPI modules

The Markov Cluster algorithm (MCL) [32] was downloaded at http://micans.org/mcl. The algorithm simulates flow on the PPI graph by constructing its adjacency matrix and computing its successive powers to increase the contrast between regions with high flow and regions with low flow. The graph was then partitioned into high-flow regions corresponding to protein modules, separated by regions of no flow.

In the MCL algorithm, the inflation coefficient (I) is the main value that affects cluster granularity. A big I value will tend to result in fine-grained clusters. We selected five I values to predict protein modules: 1.4, 1.8, 2.2, 2.6, and 3.0. Since there is no known protein complex database available for *E. coli *O157 that would allow us to identify the optimal value, we used the MCL evaluation program combined with references and finally chose I = 1.8 for further research.

The modules predicted by the MCL algorithm have no overlapping components, but in real organisms some proteins exist in multiple complexes or participate in several cellular processes at the same time. Shuye Pu et al. [77] solved this problem by a step of simple post-processing. In that step, proteins assigned to each cluster (donor cluster) were scanned for interaction partners in other clusters, and proteins interacting with a sufficiently large fraction of partners in another cluster (acceptor cluster) were also assigned to that cluster.

### Visualization

A map of predicted PPIs was drawn using Pajek software downloaded at http://vlado.fmf.uni-lj.si/pub/networks/pajek. Other PPI maps of modules were drawn by Cytoscape 2.4.0, which can be downloaded at http://www.cytoscape.org/download.php

### Other tools

The TIGR tools we used to categorize proteins can be obtained at http://www.jcvi.org/cms/research/projects/cmr/overview/. The TMpred program makes a prediction of membrane-spanning regions and their orientation. The program can be used at http://www.ch.embnet.org/software/TMPRED_form.html. SignalP predicts the presence and location of signal peptide cleavage sites in amino acid sequences from different organisms: Gram-positive prokaryotes, Gram-negative prokaryotes, and eukaryotes. It is also an online tool and can be obtained at http://www.cbs.dtu.dk/services/SignalP/

## Competing interests

The authors declare that they have no competing interests.

## Authors' contributions

XW performed the research and drafted the manuscript. LL and JY formulated the study, gave informative suggestions upon the research and refined the manuscript. XR generated the domain interaction matrix from reliable PPIs in DIP and provided some scripts in PPIs prediction. MT gave useful advice and help in programming. YW and BL participated in analysis and discussion in preparing the manuscript. All authors read and approved the final manuscript.

## Supplementary Material

Additional file 1 **Predicted PPIs dataset**. Final dataset of predicted PPIs in this study, containing 12,130 PPIs with 1652 proteins involved. Click here for file

Additional file 2 **Profile of predicted PPI map**. Green nodes represent proteins, blue edges represent interactions. Click here for file

Additional file 3 **Degree distribution of random PPIs dataset**. The random PPIs dataset follows the Poisson distribution. Click here for file

Additional file 4 **Result of shared protein**. Shared protein identified by a a post-processing step. Click here for file

Additional file 5 **Dataset of predicted modules**. Data of 172 modules predicted in this study. Click here for file

Additional file 6 **The size distribution of 172 predicted protein modules**. The biggest module contains 83 proteins, while the smallest module contains only 2 proteins. Click here for file

Additional file 7 **Enrich GO term and P-values for each predicted module**. A list of enrich GO term and P-value for each module if applicable. Click here for file

Additional file 8 **Comparison of predicted modules and conserved protein complexes**. Comparison of predicted modules with protein complexes in BOND and in published references in PubMed. Details of the 55 of our predicted modules (32.0%) have complexes conserved in other bacteria. Click here for file

Additional file 9 **Three examples of comparison with known conserved protein complexes**. A: protein interaction map of module 62. B: protein interaction map of module 114. C: protein interaction map of module 126. Click here for file

Additional file 10 **Other pathogenic related modules**. A: module 88; B: module 115; C: module 130. Click here for file

Additional file 11 **KEGG pathway map for ABC transporters**. Yellow panes represent proteins which have overlap with predicted module 4. Click here for file
