# Supplementary figures and images for: Modularity analysis based on predicted protein-protein interactions provides new insights into pathogenicity and cellular process of Escherichia coli O157:H7
Source: Theor Biol Med Model. 2011 Dec 22;8:47. doi: 10.1186/1742-4682-8-47 (PMC3275473; doi:10.1186/1742-4682-8-47)

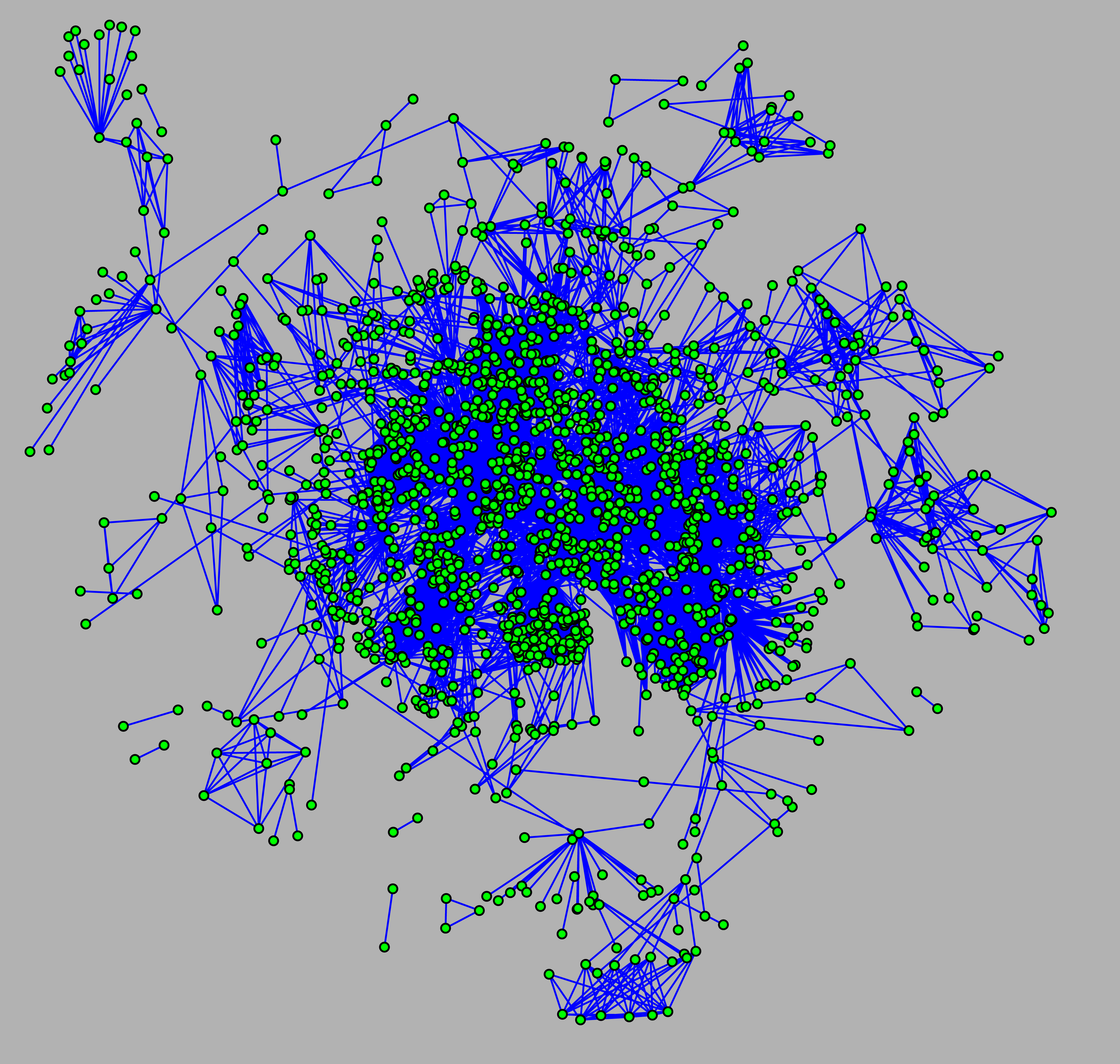

Supplement: Additional file 2 — Profile of predicted PPI map. Green nodes represent proteins, blue edges represent interactions. [file 1742-4682-8-47-S2.TIFF]

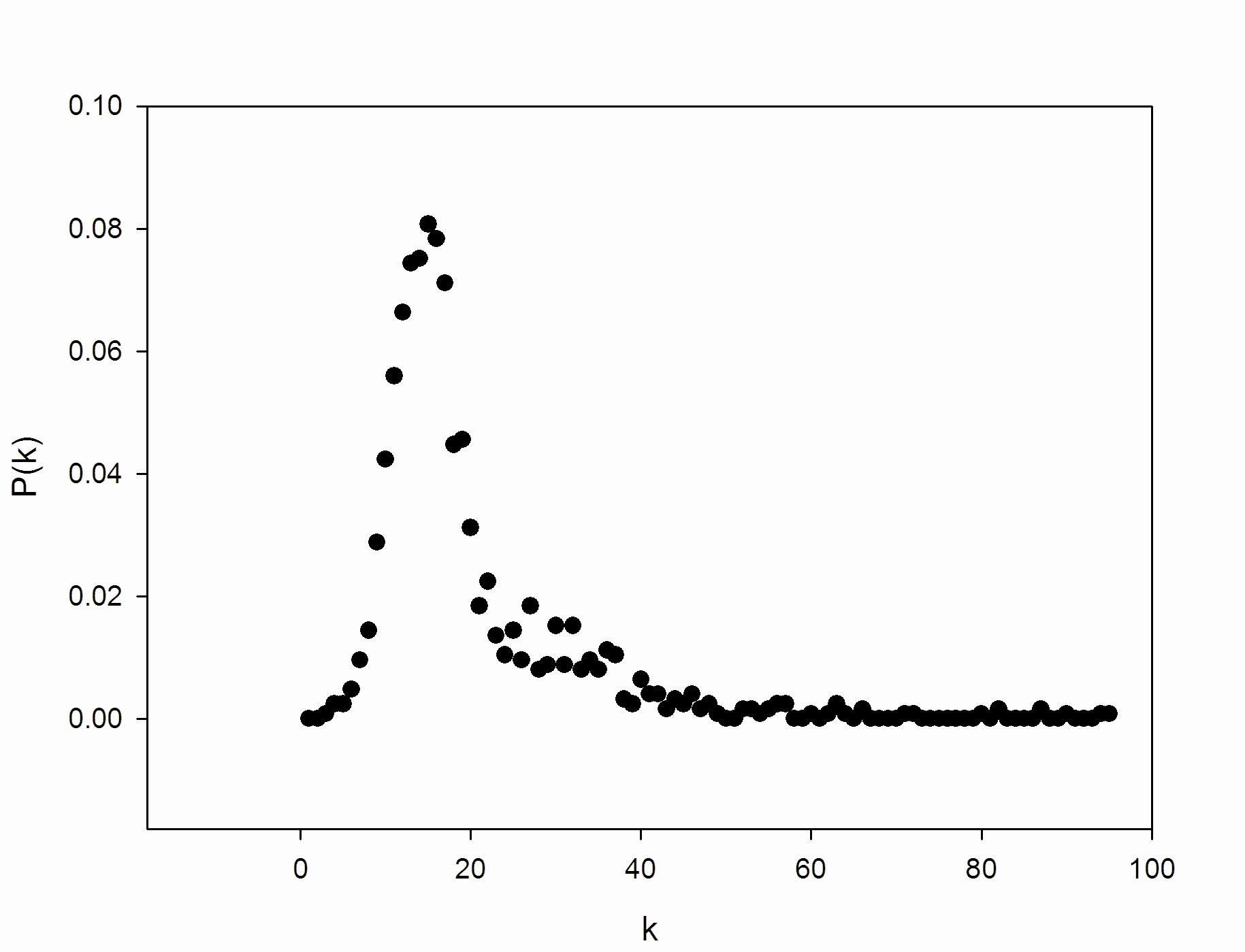

Supplement: Additional file 3 — Degree distribution of random PPIs dataset. The random PPIs dataset follows the Poisson distribution. [file 1742-4682-8-47-S3.TIFF]

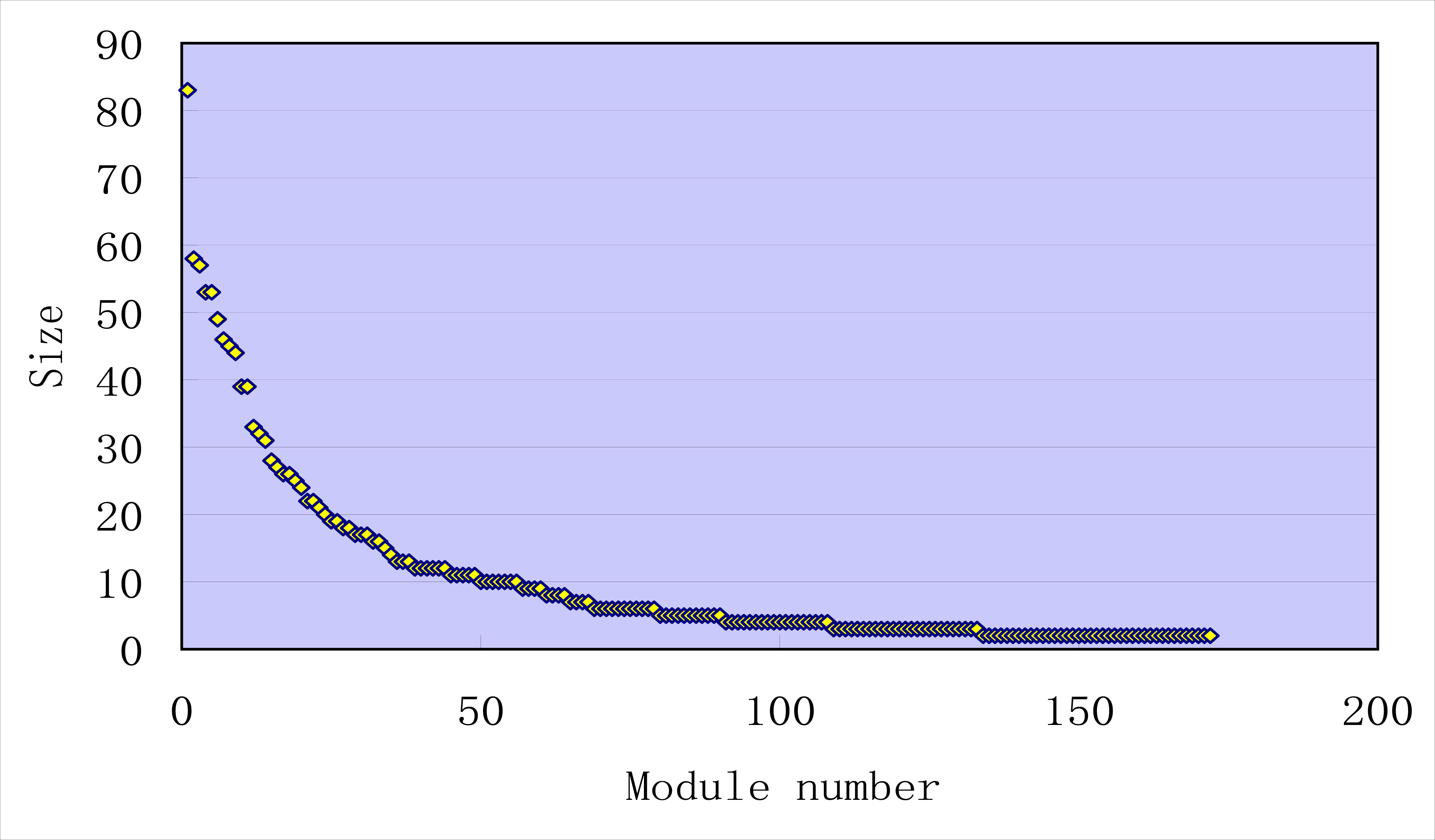

Supplement: Additional file 6 — The size distribution of 172 predicted protein modules. The biggest module contains 83 proteins, while the smallest module contains only 2 proteins. [file 1742-4682-8-47-S6.TIFF]

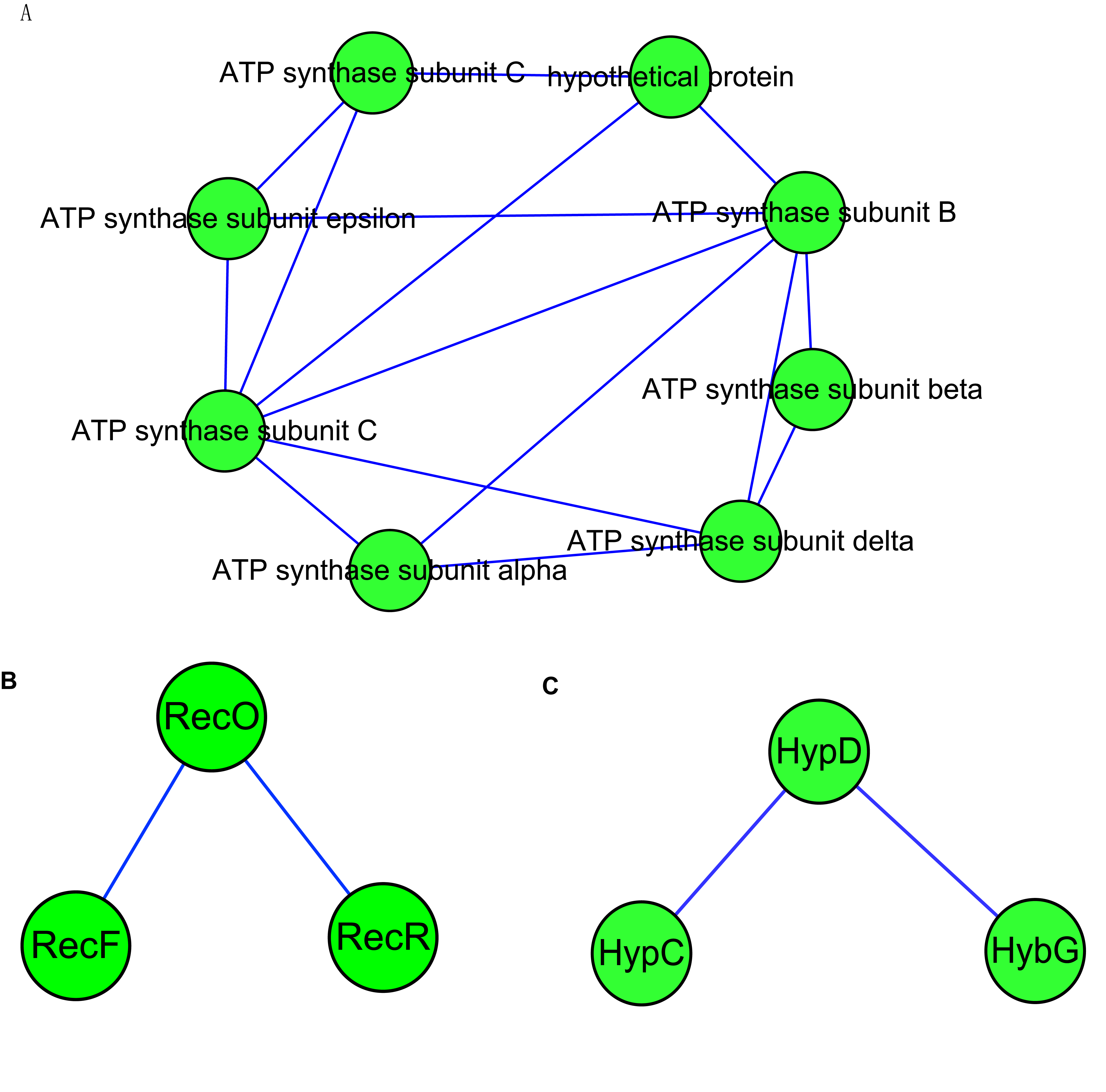

Supplement: Additional file 9 — Three examples of comparison with known conserved protein complexes. A: protein interaction map of module 62. B: protein interaction map of module 114. C: protein interaction map of module 126. [file 1742-4682-8-47-S9.TIFF]

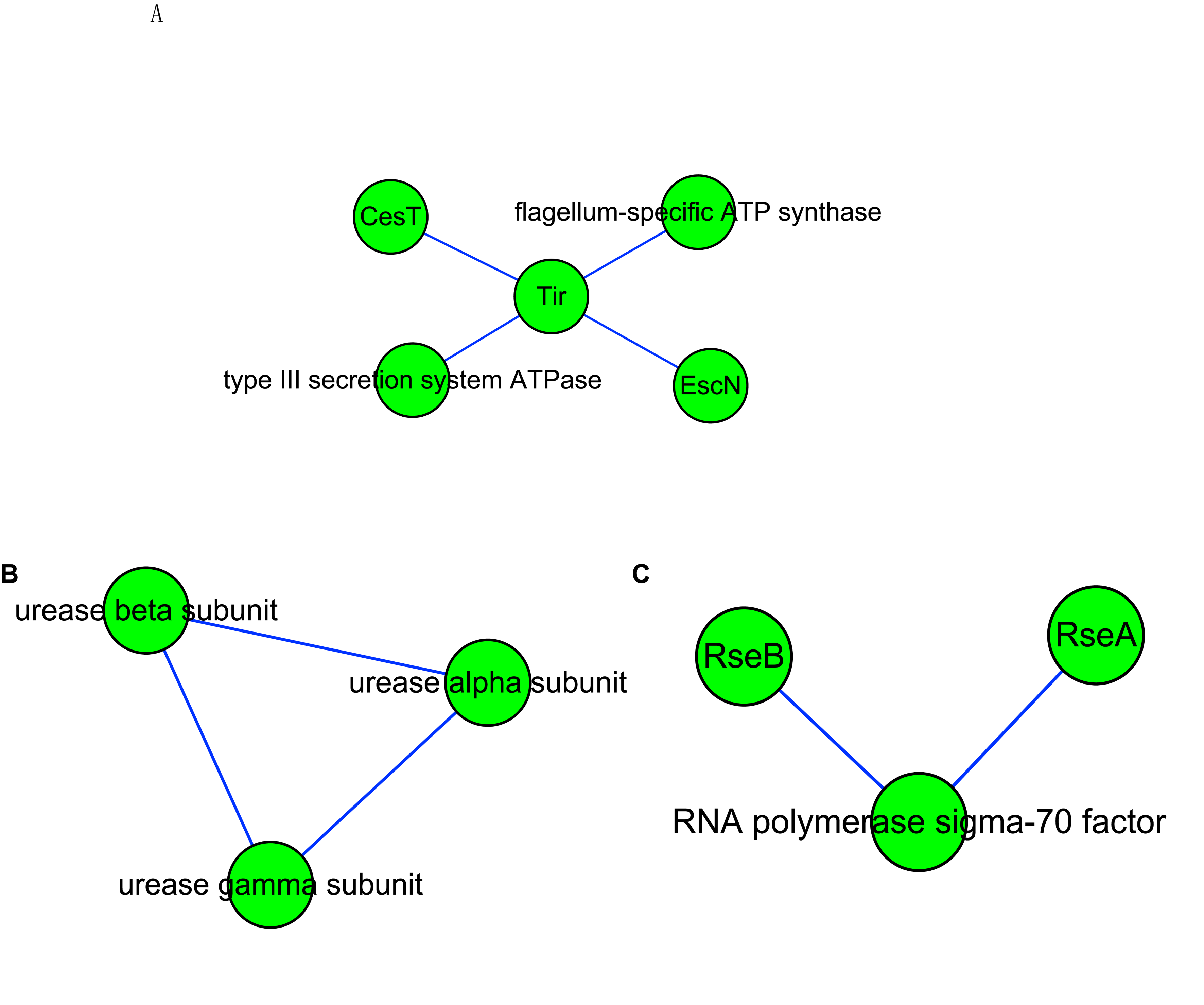

Supplement: Additional file 10 — Other pathogenic related modules. A: module 88; B: module 115; C: module 130. [file 1742-4682-8-47-S10.TIFF]

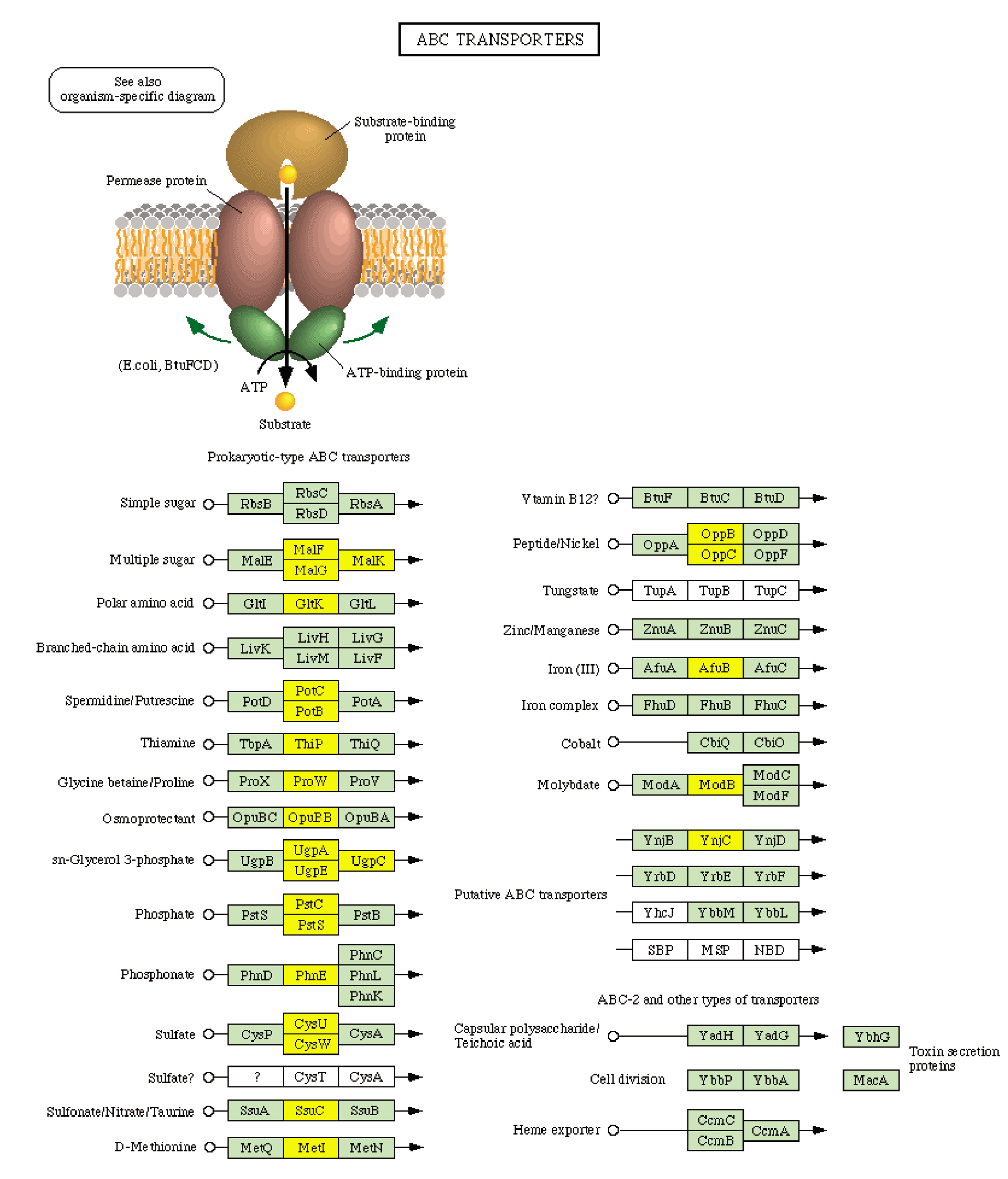

Supplement: Additional file 11 — KEGG pathway map for ABC transporters. Yellow panes represent proteins which have overlap with predicted module 4. [file 1742-4682-8-47-S11.TIFF]
