# Supplementary material for: Modularity analysis based on predicted protein-protein interactions provides new insights into pathogenicity and cellular process of Escherichia coli O157:H7
Source: Theor Biol Med Model. 2011 Dec 22;8:47. doi: 10.1186/1742-4682-8-47 (PMC3275473; doi:10.1186/1742-4682-8-47)
Supplement: Additional file 4 — Result of shared protein. Shared protein identified by a a post-processing step. [file 1742-4682-8-47-S4.PDF]

Table S1. The result of shared proteins

Shared protein	Overlapped modules	Percentage of overlapped module	Avg. protein per overlapped module
115	33	19.2%	3.48
