# Supplementary material for: Modularity analysis based on predicted protein-protein interactions provides new insights into pathogenicity and cellular process of Escherichia coli O157:H7
Source: Theor Biol Med Model. 2011 Dec 22;8:47. doi: 10.1186/1742-4682-8-47 (PMC3275473; doi:10.1186/1742-4682-8-47)
Supplement: Additional file 7 — Enrich GO term and P-values for each predicted module. A list of enrich GO term and P-value for each module if applicable. [file 1742-4682-8-47-S7.PDF]

Table S1. Enrich GO term and P-values for each predicted module

Module ID	Size	P-value	Enriched Go term
#1	83	2.52E-26	transcription
#2	58	5.92E-03	two-component signal transduction system (phosphorelay)
#3	57	4.40E-04	M phase
#4	53	5.66E-28	transport
#5	53	3.41E-34	transcription
#6	49	1.86E-37	biological adhesion
#7	46	2.82E-29	two-component signal transduction system (phosphorelay)
#8	45	3.46E-23	signal transduction
#9	44	1.14E-03	DNA-dependent DNA replication
#10	39	1.87E-13	RNA processing
#11	39	1.42E-08	gene expression
#12	33	2.97E-03	biological regulation
#13	32	6.13E-33	DNA integration
#14	31	7.94E-22	protein secretion
#15	28	1.15E-04	response to stress
#16	27	1.77E-10	translation
#17	26	6.17E-20	peptidyl-histidine phosphorylation
#18	26	2.72E-09	cellular polysaccharide biosynthetic process
#19	25	1.42E-13	oxidation reduction
#20	24	NULL	NULL
#21	22	1.39E-06	oxidation reduction
#22	22	5.79E-10	cellular polysaccharide biosynthetic process
#23	21	3.30E-13	DNA replication
#24	20	7.39E-16	transcription, DNA-dependent
#25	19	1.01E-02	regulation of nucleobase, nucleoside, nucleotide and nucleic acid metabolic process
#26	19	4.77E-03	protein folding
#27	18	6.56E-09	regulation of transcription, DNA-dependent
#28	18	1.02E-02	oxidation reduction
#29	17	8.67E-11	two-component signal transduction system (phosphorelay)
#30	17	1.60E-06	DNA replication
#31	17	4.09E-02	group transfer coenzyme metabolic process
#32	16	3.67E-03	proteolysis
#33	16	5.79E-07	carbohydrate metabolic process
#34	15	4.14E-04	ion transport
#35	14	3.22E-09	siderophore transport
#36	13	2.84E-04	proteolysis

#37	13	NULL	NULL
#38	13	3.31E-11	regulation of cell shape
#39	12	1.87E-06	glutamate biosynthetic process
#40	12	2.21E-04	lipid catabolic process
#41	12	4.80E-04	phosphoenolpyruvate-dependent sugar phosphotransferase system
#42	12	1.15E-08	tricarboxylic acid cycle
#43	12	3.20E-03	response to temperature stimulus
#44	12	2.27E-04	transcription
#45	11	1.26E-07	oxidation reduction
#46	11	8.91E-03	regulation of transcription, DNA-dependent
#47	11	6.49E-04	cellular polysaccharide biosynthetic process
#48	11	9.38E-06	transcription
#49	11	9.48E-08	oxidation reduction
#50	10	2.76E-02	DNA replication
#51	10	4.39E-02	protein refolding
#52	10	5.53E-07	proteolysis
#53	10	1.22E-04	GDP-mannose metabolic process
#54	10	1.98E-03	glucose metabolic process
#55	10	NULL	NULL
#56	10	1.12E-06	biopolymer biosynthetic process
#57	9	3.63E-04	protein folding
#58	9	1.63E-02	DNA mediated transformation
#59	9	4.09E-03	amino sugar metabolic process
#60	9	2.12E-03	amino acid metabolic process
#61	8	8.00E-07	cobalamin metabolic process
#62	8	1.67E-16	energy coupled proton transport, down electrochemical gradient
#63	8	3.54E-02	glutamyl-tRNA aminoacylation
#64	8	6.23E-17	cell redox homeostasis
#65	7	7.06E-10	phosphoenolpyruvate-dependent sugar phosphotransferase system
#66	7	1.23E-09	DNA repair
#67	7	3.95E-02	pyrimidine ribonucleoside metabolic process
#68	7	NULL	NULL
#69	7	2.40E-06	protein folding
#70	6	1.04E-04	cell division
#71	6	NULL	NULL
#72	6	6.12E-08	cell redox homeostasis
#73	6	NULL	NULL
#74	6	7.76E-05	transcription
#75	6	2.23E-06	establishment of protein localization
#76	6	3.90E-02	carbohydrate phosphorylation
#77	6	4.91E-03	regulation of transcription, DNA-dependent
#78	6	NULL	NULL
#79	6	1.43E-04	cell redox homeostasis
#80	5	7.61E-06	carnitine metabolic process

#81	5	7.28E-05	nucleotide-excision repair
#82	5	NULL	NULL
#83	5	4.46E-03	transcription
#84	5	5.19E-03	response to xenobiotic stimulus
#85	5	2.85E-08	lipopolysaccharide metabolic process
#86	5	3.18E-02	proteolysis
#87	5	5.28E-04	tRNA processing
#88	5	3.75E-04	protein secretion by the type III secretion system
#89	5	1.31E-03	RNA methylation
#90	5	5.70E-03	oxidation reduction
#91	4	1.75E-02	translation
#92	4	6.86E-05	translation
#93	4	6.27E-06	peptidoglycan-based cell wall organization and biogenesis
#94	4	9.04E-06	intracellular protein transport across a membrane
#95	4	NULL	NULL
#96	4	3.91E-09	intracellular protein transport across a membrane
#97	4	9.81E-05	translation
#98	4	6.42E-05	nucleotide-excision repair
#99	4	1.38E-02	thymidine metabolic process
#100	4	1.60E-05	hexose metabolic process
#101	4	1.32E-05	negative regulation of cellular protein metabolic process
#102	4	1.59E-05	spermidine transport
#103	4	3.61E-04	carbohydrate metabolic process
#104	4	NULL	NULL
#105	4	9.76E-03	photosynthesis, light reaction
#106	4	1.90E-04	lysyl-tRNA aminoacylation
#107	4	7.47E-07	DNA modification
#108	4	1.76E-04	DNA repair
#109	3	2.67E-06	arginine biosynthetic process
#110	3	2.04E-02	purine nucleotide metabolic process
#111	3	NULL	NULL
#112	3	4.46E-03	transcription
#113	3	7.00E-05	regulation of nitrogen utilization
#114	3	2.09E-04	DNA repair
#115	3	1.19E-05	amide metabolic process
#116	3	NULL	NULL
#117	3	2.53E-04	'de novo' pyrimidine base biosynthetic process
#118	3	9.62E-07	phospholipid biosynthetic process
#119	3	5.52E-03	modification by symbiont of host morphology or physiology
#120	3	4.55E-02	cysteine metabolic process
#121	3	3.57E-02	metallo-sulfur cluster assembly
#122	3	2.03E-03	translation
#123	3	NULL	NULL
#124	3	6.23E-03	proteolysis

#125	3	1.44E-04	proteolysis
#126	3	NULL	NULL
#127	3	1.17E-03	translation
#128	3	1.17E-03	translation
#129	3	6.04E-03	pyrimidine ribonucleoside triphosphate metabolic process
#130	3	3.76E-02	protein-DNA complex assembly
#131	3	6.23E-03	proteolysis
#132	3	2.45E-03	generation of precursor metabolites and energy
#133	3	3.12E-02	pyrimidine salvage
#134	2	9.60E-05	mRNA metabolic process
#135	2	7.94E-05	cytokinesis
#136	2	8.34E-05	NAD biosynthetic process
#137	2	NULL	NULL
#138	2	4.84E-04	fatty acid biosynthetic process
#139	2	2.85E-02	tricarboxylic acid cycle
#140	2	NULL	NULL
#141	2	NULL	NULL
#142	2	6.71E-03	negative regulation of cell division
#143	2	NULL	NULL
#144	2	8.95E-03	N-acetylglucosamine metabolic process
#145	2	1.63E-02	biotin metabolic process
#146	2	1.10E-02	fructose 6-phosphate metabolic process
#147	2	NULL	NULL
#148	2	5.96E-06	polysaccharide transport
#149	2	NULL	NULL
#150	2	NULL	NULL
#151	2	2.76E-02	translation
#152	2	NULL	NULL
#153	2	1.22E-02	indole derivative biosynthetic process
#154	2	7.28E-06	glutamine biosynthetic process
#155	2	8.13E-03	malate metabolic process
#156	2	1.06E-05	phenylalanyl-tRNA aminoacylation
#157	2	2.32E-06	trehalose biosynthetic process
#158	2	8.60E-06	tetrahydrofolate metabolic process
#159	2	NULL	NULL
#160	2	NULL	NULL
#161	2	1.65E-06	SRP-dependent cotranslational protein targeting to membrane
#162	2	3.67E-02	transcription
#163	2	NULL	NULL
#164	2	NULL	NULL
#165	2	3.14E-06	protein stabilization
#166	2	NULL	NULL
#167	2	1.08E-03	aspartate family amino acid biosynthetic process
#168	2	2.51E-04	intracellular protein transport across a membrane

#169	2	2.36E-03	transition metal ion transport
#170	2	2.08E-05	deoxyribonucleoside diphosphate metabolic process
#171	2	8.95E-03	proteolysis involved in cellular protein catabolic process
#172	2	NULL	NULL

"NULL" mean that no significant enriched GO terms are found or most of the proteins in the module are not annotated in Uniprot GOA annotation
