# Supplementary material for: Modularity analysis based on predicted protein-protein interactions provides new insights into pathogenicity and cellular process of Escherichia coli O157:H7
Source: Theor Biol Med Model. 2011 Dec 22;8:47. doi: 10.1186/1742-4682-8-47 (PMC3275473; doi:10.1186/1742-4682-8-47)
Supplement: Additional file 8 — Comparison of predicted modules and conserved protein complexes. Comparison of predicted modules with protein complexes in BOND and in published references in PubMed. Details of the 55 of our predicted modules (32.0%) have complexes conserved in other bacteria. [file 1742-4682-8-47-S8.PDF]

**Table** S1 **Comparison of predicted modules and conserved protein complexes**

| Moudle ID | description |
|---|---|
| #23 | DNA polymerase Ⅲ |
| #35 | TonB dependent transport system |
| #38 | complex related to cell division |
| #42 | catalyzing the interconversion of fumarate and succinate |
| #43 | complex related to heat-shock response |
| #46 | ccd addiction system |
| #54 | BIND ID 208731 |
| #61 | complex related to cell division |
| #62 | ATP synthase |
| #64 | disulfide bond formation system |
| #70 | complex related to cell division inhibition |
| #75 | tolQRA complex |
| #77 | transcription regulation complex |
| #88 | Tir related transport complex |
| #90 | BIND ID 208755 |
| #91 | 30S ribosome subunit of 70S ribosome |
| #93 | cell wall biosynthesis |
| #94 | Sec translocation system |
| #96 | Tat transport system |
| #97 | 50S ribosome subunit of 70S ribosome |
| #98 | BIND ID 208581、208560 |
| ＃100 | transaldolase |
| #101 | shiga toxin |
| #104 | BIND ID 208765 |
| #105 | BIND ID 208777 |
| #106 | BIND ID 208656 |
| #107 | type I restriction-modification enzyme |
| #108 | involved in recombinational DNA repair |
| #109 | BIND ID 208390 |
| #110 | BIND ID 208575 |
| #113 | GlnKBL complex |
| #114 | RecORF complex |
| #115 | urease |
| #119 | BIND ID 208549 |
| #120 | cysteine desulfurase complex |
| #122 | 50S ribosome subunit of 70S ribosome |
| #126 | HybCDG complex |
| #125 | peptidase |

| | |
|---|---|
| #127 | 50S ribosome subunit of 70S ribosome |
| #128 | 50S ribosome subunit of 70S ribosome |
| #130 | sigmaE related signal transduction complex |
| #132 | dihydrolipoamide acetyltransferase |
| #135 | complex related to cell division |
| #138 | acetyl-CoA carboxylase |
| #139 | citrate synthase |
| #142 | lon related complex |
| #155 | malic enzyme |
| #156 | phenylalanyl-tRNA synthetase |
| #161 | Sec translocation system |
| #163 | DNA helicase |
| #164 | methinine biosynthesis |
| #165 | CbpAB complex |
| #167 | aspartokinase |
| #168 | Sec translocation system |
| #171 | HslUV complex |
